# CD44 Variant Exon 6 Isoform Expression as a Potential Predictor of Lymph Node Metastasis in Invasive Breast Carcinoma of No Special Type

**DOI:** 10.1155/2021/1586367

**Published:** 2021-12-10

**Authors:** Primariadewi Rustamadji, Elvan Wiyarta, Kristina A. Bethania

**Affiliations:** ^1^Department of Anatomic Pathology, Faculty of Medicine, Universitas Indonesia, Jakarta, Indonesia; ^2^Faculty of Medicine, Universitas Indonesia, Jakarta, Indonesia

## Abstract

**Background:**

Invasive breast carcinoma of no special type (IBC-NST) is the most widespread invasive carcinoma subtype causing primarily regional metastases of the lymphatic node (LNM). The capacity of CD44 variant exon 6 (CD44v6) expression as an LNM predictor biomarker in IBC-NST was explored.

**Methods:**

We conducted a cross-sectional research with 48 paraffin blocks containing IBC-NST primary tumors that were divided into two groups by LNM. The assessment has been carried out in terms of age, tumor size, tumor grade, lymphovascular invasion (LVI), and CD44v6 expression. The expression of CD44v6 was analyzed on the grounds of immunohistochemical (IHC) staining, while other data were taken from archives. Statistical analysis is carried out by univariate, multivariate, and AUROC.

**Results:**

CD44v6 exhibits a dominant expression in IBC-NST tumor cells. Univariate analysis revealed a significant association between CD44v6 and LNM status (*p* = 0.001). Multiple logistic regression results showed that CD44v6 expression and LVI were significantly associated with LNM with OR 10.7 (95% CI: 2.43 to 47.08) and 6.22 (95% CI: 1.4 to 27.88), respectively. CD44v6 expression was able to discriminate against LNM with AUROC 0.863 ± 0.053 (95% CI: 0.759 to 0.967) at the H-score cut-off 133.889 (75% sensitivity and 83.3% specificity).

**Conclusion:**

CD44v6 expression and LVI are potential predictors of LNM in IBC-NST. The H-score cut-off of the CD44v6 expression can also be used as a threshold for classification in further investigation.

## 1. Introduction

Breast cancer is the most prevalent and lethal form of cancer in women. Twenty-five percent of cancers that occur in women are breast cancers. The incidence of breast cancer globally hit 2.09 million new cases in 2020 [1]. According to the Global Cancer Incidence, Mortality and Prevalence (GLOBOCAN) records, breast cancer mortality has reached 626,679 cases [1]. In general, breast cancer is a tumor that is caused by the unregulated progression of breast tissue. This unregulated progression is due to several causes, such as internal factors (age, genetic, hormone, etc.) or external factors (diet, insufficient exercise, obesity, etc.) [2]. In order to promote the diagnosis of cancer, the histological classification of breast cancer is classified into two groups, depending on their connection to the basement membrane, namely, noninvasive and invasive carcinoma [3, 4]. Both of these forms have subtypes of their own. Of the invasive subtypes of carcinoma, 80% are invasive ductal carcinoma of no special type (IBC-NST) [5].

The worse the grading of a breast cancer, the worse the prognosis of the patient [6, 7]. This is because the risk of metastases would be higher in high-grade carcinomas, e.g., IBC-NST [8]. Metastasis is a stage of cancer development that may cause cancer cells to migrate and develop in other organs [9]. Metastasis occurs by developing new cancer cell colonies in other organs, further worsening the patient's prognosis [9]. In IBC-NST, the most common organ for metastases is axillary lymph node, hereinafter referred to as lymph node metastases (LNM) [10].

Numerous attempts have been made to predict LNM, in particular for the IBC-NST subtype [11]. One of these is the detection of unique biomarkers in IBC-NST [11]. Researchers have so far managed to identify different biomarkers unique to the IBC-NST subtype, namely, collagen, AKT2, etc. [11, 12]. In this study, we see the potential of CD44v6 as LNM predictors in IBC-NST. CD44 is one of the proteins that play a part in the pathway of cancer invasion and epithelial-mesenchymal transformation (EMT) influencing the patient's prognosis [13]. CD44 consists of 2 isoforms after undergoing alternative splicing, namely, standard isoform (CD44s) and variant isoform (CD44v) [14]. It has been hypothesized that CD44v overexpression has a role in the development of some malignancies [15]. There are several types of exon variants in CD44v, but the CD44 with variant exon 6 (CD44v6) isoform has received the most attention [15]. Several studies have shown a significant relationship between CD44 and invasive breast cancer, although they were using advanced molecular methods, which is not generally practical, and did not specifically describe investigating the role of CD44v6 in IBC-NST [4, 16, 17]. On the other hand, some studies have even shown that there is no relationship between CD44 and invasive breast cancer [18–20]. Moreover, Umeda et al. [21] found that CD44v6 expression showed no relationship with LNM in IBC-NST; however, this study had a limited sample size and did not focus on the axillary lymph nodes.

These controversial results challenged us to participate in research on CD44 in invasive breast cancer, especially identifying expression of CD44v6 in IBC-NST lymph node metastasis using immunohistochemistry, a practical method that is also quite reliable. We also determine the optimal cut-off for the CD44v6 H-score (expression score in IHC staining) so that it is expected to be used as a data separation guidance for IHC staining in further IBC-NST-related research.

## 2. Materials and Methods

### 2.1. Study Design and Data Collection

This research is a cross-sectional study performed in the Anatomical Pathology Laboratory, Faculty of Medicine, University of Indonesia from September 2020 to January 2021. The experimental protocols number 20-09-1169 have been accepted by the Ethics Committee of the Faculty of Medicine, University of Indonesia. The research was performed with the knowledge and informed permission of each participant and in compliance with the Code of Ethics of the World Medical Association (Declaration of Helsinki) [22]. Any details are recovered from the departmental archives of 2019. The data obtained were patient age, tumor subtype, tumor category, tumor size, LVI, and LNM. All of the patients undergo mastectomy in 2019. CD44v6 expression data were obtained from the quantification in immunohistochemistry staining.

### 2.2. Samples

The sample of this study were primary tumor paraffin blocks from Asian female breast mastectomy in 2019 which has been firstly diagnosed histopathologically with IBC-NST, either with or without metastases in their adjacent lymph node. Samples having an additional histopathological diagnosis other than the IBC-NST (e.g., invasive lobular carcinoma, medullary carcinoma, and papillary carcinoma), having systemic comorbidities (hypertension, diabetes mellitus, etc.), or samples with unreliable paraffin blocks (e.g. broken/damaged paraffin blocks and paraffin blocks whose tumor mass is cut or eaten by animals) were excluded.

Samples were categorized on the basis of the presence or absence of LNM with the minimum of 23 samples for each group. In this analysis, we used 24 samples in the LNM group and 24 samples in the non-LNM group. In order to avoid biases, only one researcher (E.W.) was able to access the grouped data. Other researchers do not know which group their sample belongs to until the experiment is done.

### 2.3. Slide Preparation and Immunohistochemistry Staining

By cutting the tissue from a paraffin block using a microtome of 3-5 *μ*m thickness, slide preparation was carried out, preparation followed by heating at 58°C on a slide heater for 60 minutes, deparaffinization with stratified xylol (Merck, Jakarta, Indonesia) for 5 minutes each, then followed by alcohol rehydration and water rinsing. Using 0.1 M NaOH citrate buffer (pH 7.0) in an autoclave at 121°C for 15 minutes, each slide was pretreated with heat-induced recovery antigen, then washed in phosphate buffer saline (pH 7.4) for 5 minutes. In addition, blocking was done at room temperature for 30 minutes using hydrogen peroxide in 3% *v*/*v* methanol (Brataco Inc., Jakarta, Indonesia). It was then washed for 5 minutes in running water, followed for 15 minutes in nonspecific protein blocking with Background Sniper Universal (Abcam, Jakarta, Indonesia). The slides were incubated for 1 hour with an anti-CD44v6 antibody (Abcam, Jakarta, Indonesia) with a dilution of 1 : 100 after the blocking process and then washed in PBS for 5 minutes. The slides then were incubated for 30 minutes with a biotinylated secondary antibody (Abcam, Jakarta, Indonesia) and then washed for 5 minutes again with PBS (Brataco Inc., Jakarta, Indonesia). On the slide, Diamino Benzidine Tetrahydrochloride (DAB) was dropped and counterstained for 2 minutes with Lilie Mayer's Hematoxylin (Abcam, Jakarta, Indonesia). The slides were subsequently immersed for 2 minutes in lithium carbonate (Abcam, Jakarta, Indonesia), followed by graded alcohol dehydration (each for 5 minutes) and graded xylol clearing (each for 5 minutes). Finally, liquid cover, which is aqueous mounting media, was used to cover the sections. Subsequently, the stained sections were studied for CD44v6 expression. For each stain, negative control and positive control were also included. By eliminating the primary antibody administration step, negative control was carried out.

### 2.4. Quantification of CD44v6 Expression

Two researchers (P.R. and K.A.B.) who are specialized in reading histopathological slides have performed the examination and reading of immunohistochemical stainings. Using a light microscope with a total magnification of 400x, each preparation was analyzed and recorded using a computer with Leica LAZ EZ software and a camera combined with a Leica DM750 microscope. At least 500 tumor cells from 5 separate high-power visual fields (400x) that were randomly picked were evaluated for CD44v6 expression. There were at least 100 tumor cells representing each area. Brown staining of the tumor cell membrane reflected CD44v6 positivity [23]. Intensity of staining was classified into no staining (0), low positive (1+), positive (2+), or high positive (3+) based on the brown color intensity assessed in each field using cell counter in ImageJ [24]. The H-score is used to quantify the CD44v6 expression [25]. Two observers (P.R. and K.A.B.) each measured the H-scores of the whole sample separately. The results of the previously assessed measurements are obtained by the statistician (E.W.) before the whole study has been analyzed in order to eliminate bias. For further analysis, the mean H-score of the two observers will be used.

### 2.5. Statistical Analysis

Prior to the review, data processing was entered into a table using Excel (2013 (Microsoft Corp., Redmond, WA, USA). Tabulated data will be evaluated using the Statistical Package for Social Sciences/SPSS version 20 (IBM Corp., Armonk, NY, USA) and visualized using GraphPad Prism 8 (GraphPad Software, Inc., La Jolla, CA, USA). In order to test the reliability of the results, the variability of the H-score between the two observators was compared with the Intraclass Correlation Coefficient (ICC). A two-way mixed average estimation of absolute agreement in the ICC model was used. ICC values were classified based on the 95% confidence interval of the ICC estimate: values less than 0.5, between 0.5 and 0.75, between 0.75 and 0.9, and greater than 0.90 are indicative of poor, moderate, good, and excellent reliability, respectively [26].

Using the median H-score as the cut-off (median split approach), the H-scores of the two observers were divided into high or low groups [27, 28]. This group represents the CD44v6 expression in each study. A bivariate analysis was conducted to compare age (<50 y.o. or ≥50 y.o.), tumor grade (high or low), tumor size (≤5 cm or >5 cm), LVI (yes or no), and expression of CD44v6 (high or low) against lymph node metastases (yes or no). The bivariate analysis component with a *p* value of less than 0.2 would be accompanied by multivariate analysis using the backward LR method in the multiple logistic regression model [29]. A *p* value below 0.05 is considered to be statistically significant. The discrimination ability of the model was accessed by the Receiver Operating Characteristic (ROC) curve area calculation. ROC is evaluated on the basis of Area Under the Curve (AUC) grouped by the following: 0.5 indicates no discrimination, 0.7 to 0.8 is considered acceptable, 0.8 to 0.9 is considered excellent, and more than 0.9 is considered outstanding [30].

Several confounding factors that may also have an association with LNM must be minimized and controlled in order to assess the predictive factor of CD44v6 expression for LNM. Through the use of inclusion and exclusion criteria, several confounding variables related to LNM, such as the patient's race, the existence of other histological diagnoses, tumor subtypes, and numerous comorbidities, were excluded [31]. Furthermore, numerous confounding variables which could not be excluded, such as age, tumor grade, tumor size, and LVI, were remained included in the study, but their association with LNM was accounted for using bivariate and regression analysis.

ROC curve of CD44v6 H-score as a potential biomarker was also analyzed by using CD44v6 H-score continuous data. Based on the highest Youden index [32] and the lowest K-index [33], the cut-off value of the CD44v6 expression was also identified. The Youden index is an index that uses the maximum vertical distance of the ROC curve from the point (*x*, *y*) on the diagonal line (chance line) [34]. The K-index, meanwhile, describes the distance to the cut-off (*x*^2^, *y*^2^) from the ideal (upper left corner) point (*x*^1^, *y*^1^) [33].

## 3. Results

### 3.1. Immunohistochemical Staining and H-Score Reliability

**Figure 1:**
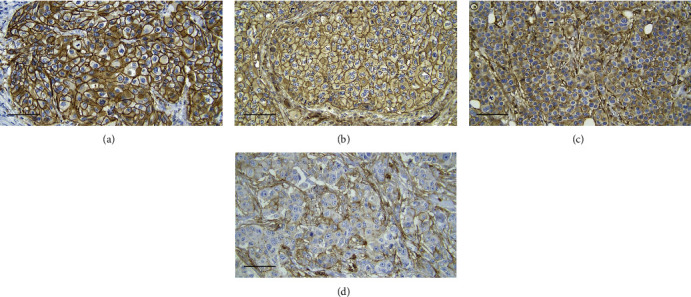
Immunohistochemical staining for CD44v6 expression in IBC-NST tumor cells at 400x magnification: (a) high positive, (b) positive (c) low positive, and (d) no staining. Scale bar represents 50 *μ*m for all images.

**Figure 2:**
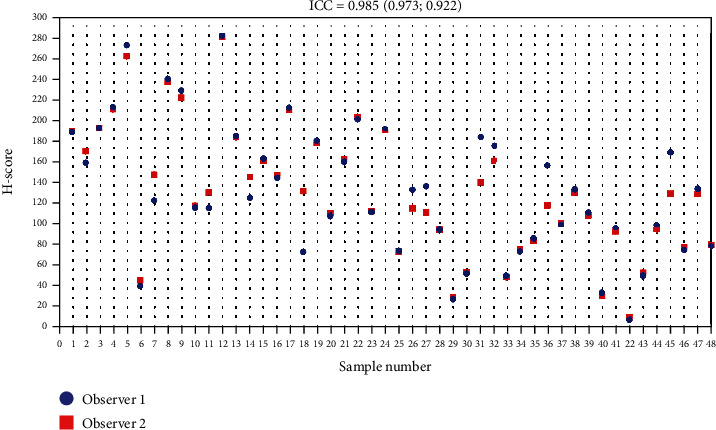
Dot plot depicting the distribution of the H-score of CD44v6 expression between two researchers over the entire sample.

All 48 specimens underwent immunohistochemical staining for the expression of CD44v6. Figures 1(a)–1(d) display the effects of representative immunohistochemical staining. Samples of tumor cells with different staining groups are given in each image, including high positive (a), positive (b), low positive (c), and no staining (d). All of these images come from the same patient. This also shows that one patient can have various types of intensity of CD44v6 expression. In the cell membrane, CD44v6 expression can be observed. In this region, the brown color intensity was quantified into an H-score for further evaluation. Two researchers (P.R. and K.A.B.) observed all 48 samples independently. The distribution of the H-score in each sample can be seen in Figure 2. An excellent reliability was found between two measurements. The average measure ICC was 0.985 with a 95% confidence interval from 0.973 to 0.992 (*F* (47.47) = 66.188, *p* < 0.001).

### 3.2. Association between CD44v6 Expression and LNM

In this study, each sample has its own clinicopathologic characteristics which can be seen in Table 1. Each of these variables is a covariate that may have a role in influencing the occurrence of LNM. Therefore, we also considered the role of each of these variables by conducting a bivariate test to see their relationship with LNM. Bivariate analysis of several variables was performed to assess their association with LNM. The variables to be tested are patient's age (<50 y.o. or ≥50 y.o.), tumor grade (grade III (high) or grade I-II (low)), tumor size (≤5 cm or >5 cm), lymphovascular invasion (yes or no), and expression of CD44v6 (high or low) which results can be seen in Table 2.

The results of bivariate analysis showed a significant relationship between CD44v6 expression and LNM (*p* = 0.001). In addition, LVI was also significantly associated with LNM (*p* = 0.02). To see the relationship between the two further, multiple logistic regression was carried out as shown in Table 3. It can be seen that both CD44v6 expression and LVI are related to LNM with odds ratios of 10.7 (95% CI: 2.43 to 47.08) and 6.22 (95% CI: 1.4 to 27.88), respectively. These results indicate that samples with high CD44v6 expression were 10.7 times more likely to have metastasis than samples with low CD44v6 expression. In addition, samples with LVI were 6.22 times more likely to have metastases than samples without the LVI. This model also calculates the metastatic probability based on the following formula [35]:

(1) $$\begin{array}{c} P\left(Y\right)=\frac{e^{X1.\left(2.37\right)+X2.\left(1.83\right)-2.22}}{1+e^{X1.\left(2.37\right)+X2.\left(1.83\right)-2.22}}. \end{array}$$

In this case, *P*(*Y*) is the probability of LNM, *X*_1_ is the CD44v6 expression (value 1 if high and 0 if low), and *X*_2_ is LVI (value 1 if yes and 0 if no). The likelihood of LNM in several situations, as can be seen in Table 4, can be determined from this model.

As seen in Table 3, the goodness of fit for the model was adequate. The Hosmer–Lemeshow test (*p* = 0.445) indicated that the numbers of LNM were not significantly different from those predicted by the model and showed a satisfactory goodness of fit. Moreover, the test of the overall model against a constant-only model was statistically significant (*p* < 0.001), indicating that the predictors as a set reliably distinguished between LNM and no LNM.

### 3.3. Receiver Operating Characteristic (ROC) of CD44v6 Expression

**Figure 3:**
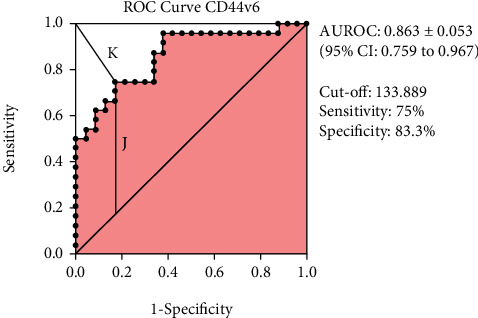
ROC curve of CD44v6 expression. J: Youden index; K: K-index.

The ability of CD44v6 expression to discriminate against lymph node metastases is also assessed through the ROC curve which can be seen in Figure 3. The area under the ROC (AUROC) was 0.863 ± 0.053 (95% CI: 0.759 to 0.967). This area shows excellent accuracy. Based on the ROC curve, the optimal cut-off point for the CD44v6 expression was 133.889 (H-score) with the highest Youden index (0.583) and lowest K-index (0.301) among others. This cut-off has 75% sensitivity and 83.3% specificity.

## 4. Discussion

We investigated the capacity of CD44v6 to predict LNM in IBC-NST in this work. According to IHC staining, CD44v6 exhibits a dominant expression in IBC-NST tumor cells. Univariate analysis revealed a significant association between CD44v6 and LNM status (*p* = 0.001). Based on multivariate analysis, CD44v6 showed the potential to be an independent factor in the prediction of LNM (OR: 10.7, 95 percent CI: 2.43 to 47.08, *p* = 0.002). In addition to CD44v6, we discovered that LVI has the potential to be an independent predictive factor (OR: 6.22, 95% CI: 1.4 to 27.88, *p* = 0.017). CD44v6 has excellent predictability with a determined optimum cut-off, according to AUROC analysis.

A variety of approaches, from basic to advance, can be used in defining CD44v6 as a cancer biomarker. Several modern techniques like the use of molecular methods in laboratories with advanced facilities are frequently used for research. However, it is less convenient to use this approach in laboratories with limited facilities. Therefore, in this research, we employed a comparatively comfortable and inexpensive method of recognizing CD44v6, i.e., IHC staining. Selective antibody was used to bind to CD44v6 in tumor cells [36]. In this study, the specific antibody binds to CD44v6 in the cell membrane. DAB is used as a chemical agent to facilitate identification of CD44v6. Activated DAB will be visualized as a brown color. As has been explained, CD44v6 attaches to the surface of the cell membrane. Therefore, the brown color more would be observed in the membrane of IBC-NST tumor cells, as shown in Figure 1. This finding is in line with the study of Xu et al. which shows that CD44 was mainly detected on the membrane of breast cancer cells [23]. This brown color identity also represents the expression level of CD44v6 in the cell membrane. The vast amount of CD44v6 molecules in the cell membrane would also activate more DAB, creating a higher intensity of the brown color. This process is the basis of the H-score quantification in this study.

CD44v6 expression with LNM had a very strong association (OR: 10.7, 95% CI: 2.43 to 47.08, *p* = 0.002) based on the multiple logistic regression model. This suggests that high CD44v6 expression in IBC-NST tumors had 10.7 times more risk of having LNM than IBC-NST tumors which had low CD44v6 expression. This result is consistent with the molecular mechanism of CD44v6, which has been demonstrated to increase the ability of invasive breast cancer cells to multiply, disseminate, and invade [37, 38]. CD44v6 has the capacity to activate c-Met, which enables the Ras-SOS signaling cascade to be activated, resulting in cell proliferation [38], and also stimulates VEGFR-2 activity, resulting in metastasis [39]. Several other pathways are also capable of being activated by the CD44v in breast cancer, for example, CD44v6 induce hypoxia-inducible factor 1*α* (HIF1*α*) so as to increase rate of glycolysis [14]. Apart from being in line with the theory that has been developed, the findings of this study are also in line with several other primary studies. Kaufmann et al. [40] found that CD44v6 is an independent marker for predicting LNM in primary breast cancer. Günthert et al. [41] found that transfection of tumor cells with CD44v6 could enhance LNM. However, some studies have shown the opposite result. Despite the fact that the patients' survival rate was not assessed, Ricardo et al. [42] show that CD44 failed to reach statistically significant levels in predicting disease-free survival and overall survival. Other similar studies from Umeda et al. [21] also found that CD44v6 expression had no connection with lymph node metastases in IBC-NST patients. However, the number of Umeda et al. [21] research samples was half that of ours, reducing the power of their study. Furthermore, not all of their samples were obtained from the axillary lymph node, but some were obtained from the mediastinal lymph node as well.

LVI is also shown to have association with LNM (OR: 6.22, 95% CI: 1.4 to 27.88, *p* = 0.017). This suggests that IBC-NST primary tumors with LVI are 6.22 times more likely to have LNM than IBC-NST tumors without LVI. The role of LVI in IBC-NST is still under research, but several researchers, such as Melzer et al. suspect the role of MMP in inducing LVI [43]. Schoppmann et al. also showed a role for LVI in inducing LNM [44]. Nathanson et al. even show that LVI plays a role in systemic metastases [45]. These findings suggest a role for LVI in inducing LNM. To our knowledge, no study has examined CD44v6 and LVI expression together. Therefore, this study can be one of the bases for understanding the principle of CD44v6 as a biomarker when found together with LVI as shown in formula (1) and Table 3. However, studies with more diverse biomarkers are needed to explore the potential for better prediction of LNM in IBC-NST.

CD44v6 has an excellent accuracy with AUROC 0.863 ± 0.053 (95% CI: 0.759 to 0.967). Moreover, we also determined the potential cut-off for the H-score of the CD44v6 expression. We conducted this process because we were aware that the use of the marker-specific IHC cut-off assay is important for the prediction of therapeutic response [46]. The use of the median H-score, like the one we used in this study, may be less specific in a larger and more variable dataset. Therefore, we identified a potential H-score cut-off for the CD44v6 greater than or equal to 133.889 with the highest Youden index (0.583) and the lowest K-index (0.301) among others. This potential cut-off has 75% sensitivity and 83.3% specificity. This means that the use of this cut-off is expected to be able to help classify high-low CD44v6 expressions and improve their discriminatory abilities.

This study has several limitations. Despite the fact that we have tried to maximize the number of samples that can be analyzed in our facility, larger sample size studies with more diverse biomarkers are still needed, indicating the possibility of other biomarkers being integrated in the prediction of ALNM on IBC-NST. Moreover, because this study was done in a tertiary hospital, the IBC-NST were mostly high-grade cancers. By examining low-grade cancers, additional research is needed to cover a more diverse population.

## 5. Conclusions

CD44v6 expression together with LVI can be used as a predictor biomarker to disseminate LNM in IBC-NST tumors. In addition, an H-score of 133,889 was also identified as a potential cut-off which could be used as a guideline for classifying the level of expression of CD44v6 in future studies.

## Figures and Tables

**Table 1:** Frequency distribution based on clinicopathological characteristics.

Clinicopathological characteristic	*N* or value	%
Age (years)		
≥50	27	56.25
<50	21	43.75
Mean (SD)	50.94 (12.29)	
Median (Min-Max)	50 (29-75)	
Tumor grade		
Grade I	5	10.4
Grade II	16	33.3
Grade III	27	56.3
Tumor size (cm)		
<2	2	4.2
2-5	28	58.3
>5	18	37.5
Lymphovascular invasion		
Yes	27	56.25
No	21	43.75

**Table 2:** The results of bivariate analysis on several variables for LNM in IBC-NST.

Variables	Category	Lymph node metastasis			*p* value	OR	95% CI	
		Yes (%)	No (%)	Total			Min	Max
Age	≥50 y.o.	14 (51.9%)	13 (48.1%)	27	1	1.19	0.38	3.71
	<50 y.o.	10 (47.6%)	11 (52.4%)	21				
Tumor grade	High	15 (55.6%)	12 (44.4%)	27	0.56	1.67	0.53	5.265
	Low	9 (42.9%)	12 (57.1%)	21				
Tumor size	>5 cm	7 (38.9%)	11 (61.1%	18	0.37	0.49	0.148	1.602
	≤5 cm	17 (56.7%)	13 (43.3%)	30				
LVI	Yes	18 (66.7%)	9 (33.3%)	27	0.02^∗^	5	1.45	17.27
	No	6 (28.6%)	15 (71.4%)	21				
CD44v6 expression	High	18 (75%)	6 (25%)	24	0.001^∗^	9	2.44	33.24
	Low	6 (25%)	18 (75%)	24				

Bivariate analysis was performed using the chi-square test with continuity correlation. ^∗^*p* value less than 0.05 is considered statistically significant.

**Table 3:** Multiple logistic regression for LNM in IBC-NST.

Predictor	*β*	SE *β*	Wald's *χ*^2^	df	*p* value	*e* ^*β*^ (odds ratio)
CD44v6 expression	2.37	0.76	9.83	1	0.002	10.7
LVI	1.83	0.77	5.71	1	0.017	6.22
Constant	-2.22	0.75	8.72	1	0.003	0.108
Test			Wald's *χ*^2^	df	*p* value	
Hosmer–Lemeshow goodness-of-fit test			1.621	2	0.445	
Overall model evaluation			19.14	2	<0.001	

SE: standard error; df: degree of freedom; *e*: Euler number ≈ 2.718.

**Table 4:** Various probabilities for LNM based on model variables.

CD44v6 expression	LVI	Probability of LNM (%)
High	Yes	87.81
High	No	53.65
Low	Yes	40.24
Low	No	9.76

## Data Availability

The data used to support the findings of this study are included in the article.
